# Assessing lateral femoral condyle cartilage prior to medial UKA: MRI vs. Valgus stress radiograph

**DOI:** 10.1186/s12891-023-06802-2

**Published:** 2023-08-26

**Authors:** Xufeng Jiao, Guanglei Cao, Jiangpeng Wu, Zheng Li, Shuai An, Jiang Huang

**Affiliations:** Department of Orthopedics, Xuanwu Hospital Capital Medical University, 45 Changchun Street, Xicheng District, Beijing, 100053 China

**Keywords:** Unicompartmental knee arthroplasty, Lateral joint space width, Valgus stress radiograph, Magnetic resonance imaging, Cartilage quality

## Abstract

**Background:**

The cartilage quality of the lateral compartment needs to be clarified prior to medial unicompartmental knee arthroplasty (UKA). Valgus stress radiograph has been recommended as the preferred tool. Some studies also show that magnetic resonance imaging (MRI) has a higher diagnostic value. So, we conducted this study to compare whether valgus stress radiographic lateral joint space width (LJSW) and MRI grading can accurately reflect cartilage quality and its screening value for UKA-suitable patients.

**Methods:**

One hundred and thirty eight knees proposed for UKA were enrolled prospectively. Valgus stress radiograph was taken to measure LJSW. LJSW > 4 mm was considered normal and suitable for UKA. For weight-bearing area cartilage of lateral femoral condyle, Recht grade was assessed by MRI preoperatively. Recht grades ≤ 2 were treated as non-high-grade injuries while Recht grades > 2 were treated as high-grade injuries. Outerbridge grade was the gold standard and was assessed intraoperatively. Patients with Outerbridge grades 0–2 (non-high-grade injuries) underwent UKA, and patients with Outerbridge grades 3–4 (high-grade injuries) underwent total knee arthroplasty (TKA). The diagnostic parameters of valgus stress radiograph and MRI for the selection of UKA candidates were calculated, and receiver operating characteristic curves were drawn. *P* < 0.05 was considered significant.

**Results:**

Of 138 knees, 120 underwent UKAs, and 18 underwent TKAs. In terms of selecting UKA candidates, the sensitivity was close between MRI (95.0%) and valgus stress radiograph (96.7%), and the specificity, accuracy, positive predictive value and negative predictive value of MRI (94.4%, 94.9%, 99.1%, 73.9%, respectively) were higher than that of valgus stress radiograph (5.9%, 85.5%, 88.0%, 20.0%, respectively). The difference in area under the curve (AUC) between MRI (0.950) and LJSW (0.602) was significant (*P* = 0.001).

**Conclusion:**

Compared with valgus stress radiograph, MRI has excellent evaluation value in diagnosing lateral weight-bearing cartilage injuries and can be used as a reliable tool for selecting suitable UKA patients.

## Background

Osteoarthritis is the most common form of arthritis and the leading cause of disability in the elderly [1–3]. Knee osteoarthritis (KOA) accounted for 83% of all osteoarthritis [4]. Unicompartmental knee arthroplasty (UKA), as an essential part of the stepwise treatment of KOA, is a minimally invasive surgery and maximizes the preservation of the native joint, thereby avoiding or postponing total knee arthroplasty (TKA) as much as possible [5].

UKA requires intact cartilage in the contralateral compartment, while full-thickness cartilage injury is a contraindication [6]. Generally, the surgeon determines the patient’s feasibility for UKA by weight-bearing radiograph and physical examination. Valgus stress radiograph or magnetic resonance imaging (MRI) are supplements of diagnosing cartilage quality in the lateral compartment. However, which is the better choice remains controversial.

Valgus stress radiograph is widely used in clinical practice [6]. Measuring the joint space width (JSW) can monitor the progression of KOA and determine the appropriate surgical method [7]. The Oxford UKA operating manual recommends lateral joint space width (LJSW) greater than 4 mm as normal and is suitable for UKA [6]. However, some studies found that joint space width is not consistent with the structural changes of articular cartilage [8, 9]. MRI has excellent soft tissue discrimination ability, and allows grading of cartilage damage [10]. But the partial volume effect of MRI can amplify high-grade injuries or make low-grade injuries poorly visible [11].

**Fig. 1:**
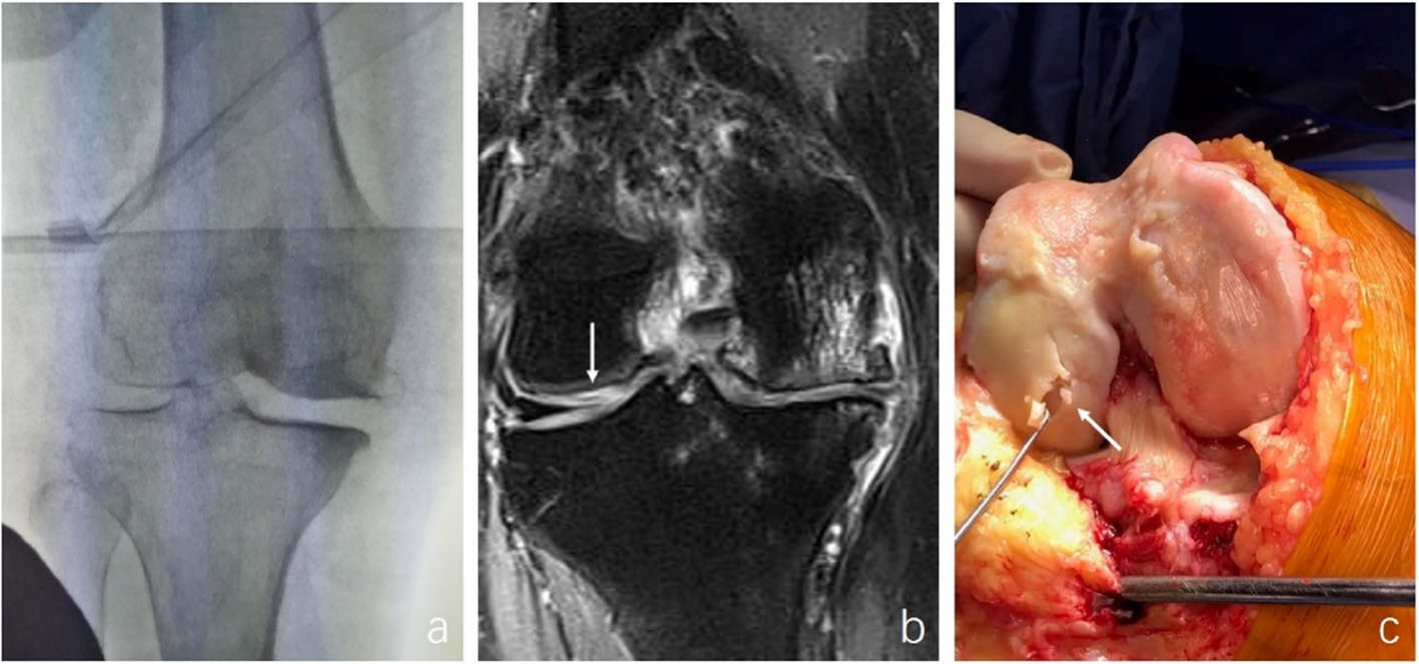
**a** A 69-year-old female whose valgus stress radiographic LJSW was normal, indicated intact articular cartilage in lateral compartmental. **b** Coronal plane on MRI showed intensive signal (arrow) in lateral femoral weight-bearing area cartilage with Recht grade 3. **c** The high-grade cartilage injury (arrow) was confirmed intraoperatively

In clinical practice, we also observed contradictory results between valgus stress radiograph and MRI, which brings confusion in surgery selection (Fig. 1). Changes in surgical plans caused by inaccurate preoperative imaging assessments reduce patients’ faith and surgeons’ confidence.

Up to now, there is no research comparing supplementary diagnostic values between MRI and valgus stress radiograph for the cartilage quality in the weight-bearing area of the lateral compartment. Although some studies have investigated the diagnostic value of valgus stress radiograph [12, 13] or MRI [11, 14–16], respectively, these studies reported sensitivities of MRI ranging from 0 to 70%, with wide variation, and specificities ranging from 78 to 100%, and the knee was not divided into medial and lateral compartments and other regions [11, 14–16]. So, it is difficult to compare the results directly due to sample selection bias by different studies. Therefore, we conducted this prospective study to compare whether valgus stress radiographic LJSW and MRI grading can accurately reflect cartilage quality in the weight-bearing area of the lateral femoral condyle and their supplementary screening value for UKA candidates.

## Methods

### Subjects

**Fig. 2:**
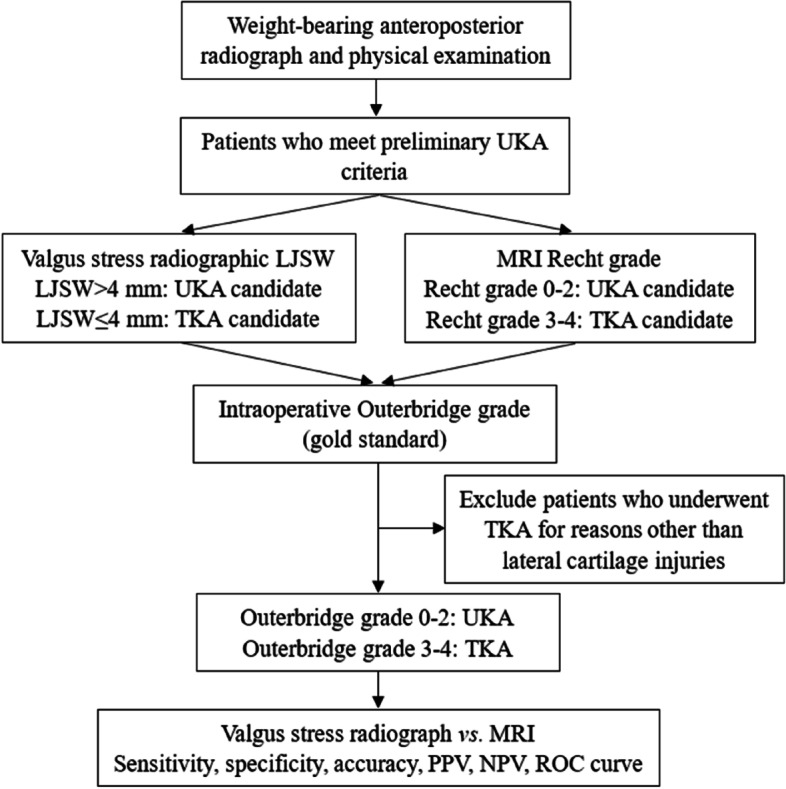
Research flow chart

This prospective study included patients with medial unicompartmental KOA and were proposed for medial UKA in our hospital between October 2018 to December 2020. The feasibility of UKA was based on the patient’s weight-bearing anteroposterior radiograph and physical examination. To be a medial UKA candidate, the following criteria must be met: weight-bearing anteroposterior radiograph showed that the medial compartment was “bone-on-bone”, and there was no significant lateral joint space narrowing; the range of motion was ≥ 90°; varus deformity was ≤ 15°; fixed flexion contracture was ≤ 15°; no patellofemoral subluxation or lateral groove-like changes; and correctable varus deformity. Both MRI and valgus stress radiograph were used to assess cartilage quality of lateral femoral condyle. Patients who eventually underwent TKA for reasons other than lateral cartilage injuries, such as anterior cruciate ligament dysfunction, were excluded. The study flow is shown in Fig. 2.

### Standard valgus stress radiograph

**Fig. 3:**
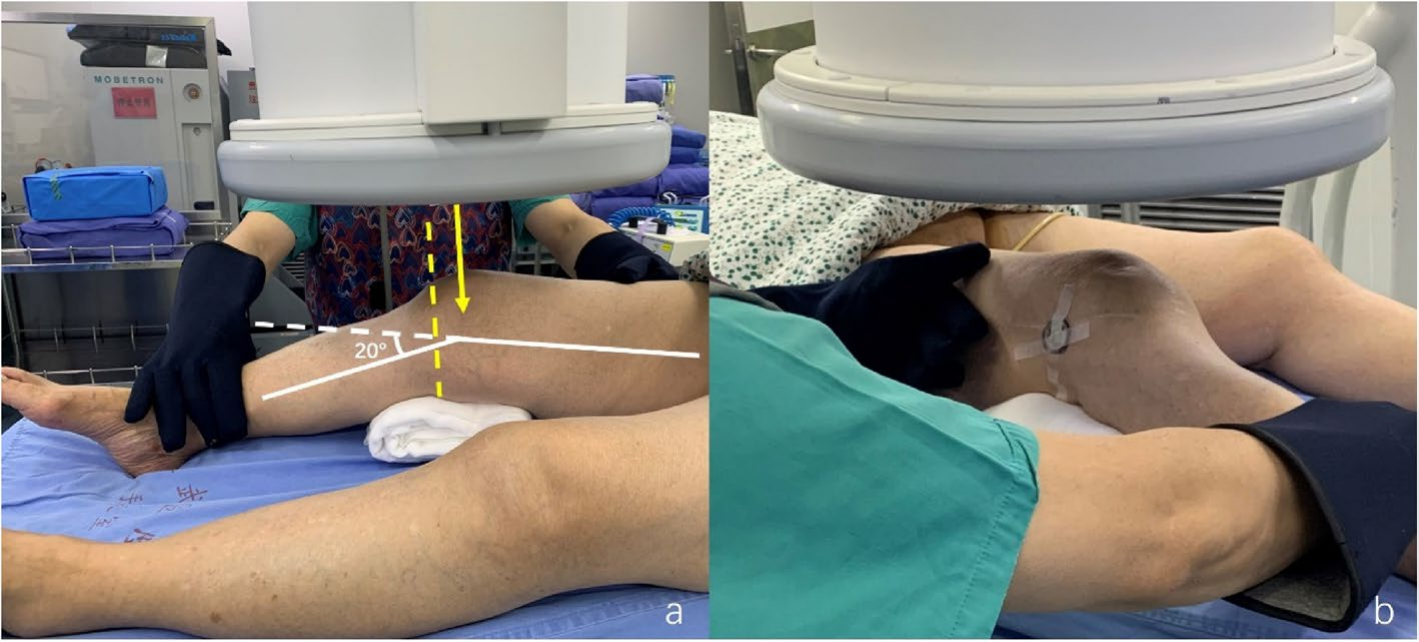
Conducting a standard valgus stress radiograph. **a** A cloth cushion was placed under the knee to form a 20° flexion. The surgeon kept the lower limb alignment neutrally and applied valgus stress manually. The X-ray beam was adjusted (yellow solid line) until it was parallel to the tibial plateau (yellow dotted line). **b** A 25 mm diameter round metal was placed in the center of the anteroposterior diameter of the distal femur to calibrate the magnification

**Fig. 4:**
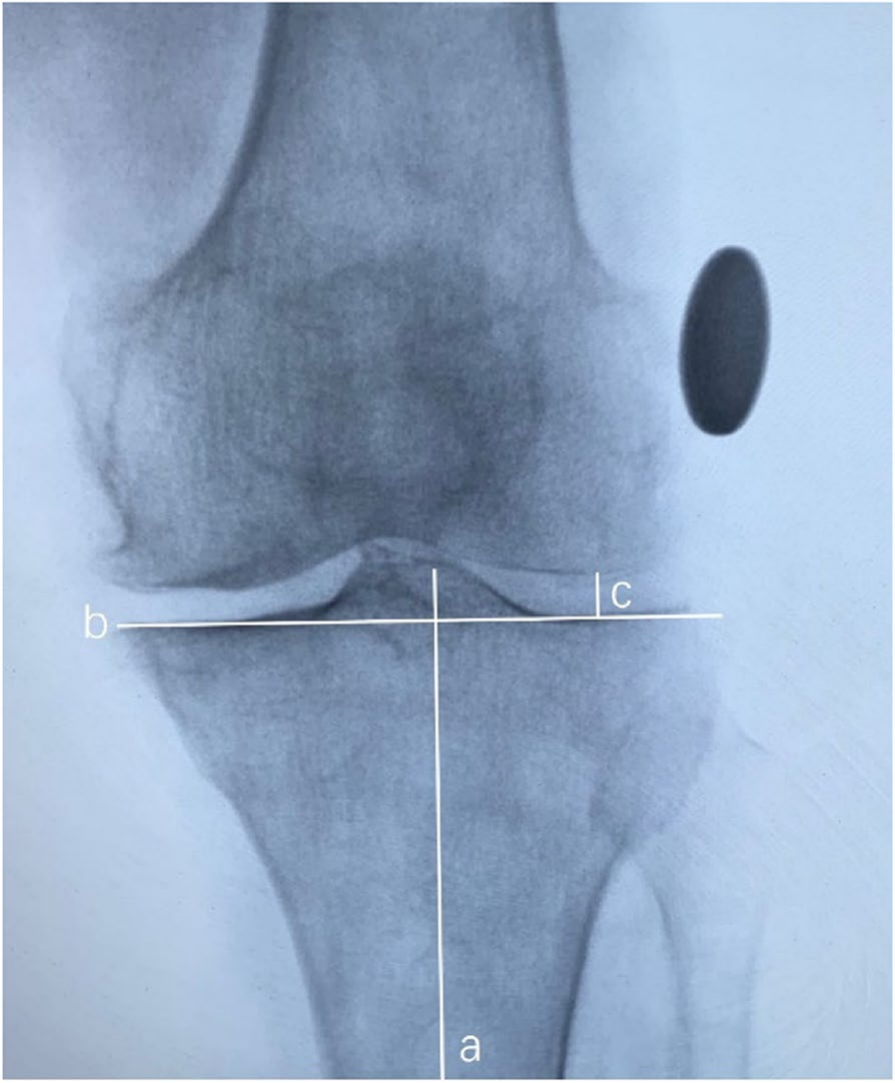
Measurement of the LJSW on the valgus stress radiograph: Line a is the tibial axis, line b is the tibial plateau cut line, and line c runs parallel to the tibial axis from the distal femoral condyle to the tibial plateau. The LJSW is identified as the length of line c calibrated by the 25 mm round metal

Standard valgus stress radiograph (General Electric Company, Boston, USA) was taken on the day of surgery. The experienced surgeon with more than 1 000 UKAs kept the lower limb alignment neutral and manually applying constant valgus stress (Fig. 3). A 25 mm diameter coin was used to calibrate for magnification. LJSW was measured by two non-operative doctors. LJSW > 4 mm was considered as UKA candidate, while LJSW ≤ 4 mm was considered abnormal and was suitable for TKA (Fig. 4).

### Magnetic resonance imaging

MRI imagining (Siemens Verio 3.0T, Munich, Germany) was obtained within two weeks preoperatively. T2-weighted fat suppression sequence images were acquired using the turbo spin-echo (TSE) pulse sequence technique (repetition time/echo time [TR/TE] = 4000/100 ms). The image thickness was 5 mm with a matrix of 256 × 256. Two doctors independently used Unisight (EBM Technologies, Inc., Taipei, China) to evaluate all patients’ weight-bearing area cartilage injury of the lateral femoral condyle in the sagittal and coronal planes, and divided the injuries by Recht grade [17] (Table 1). Recht grades 0–2 were combined as non-high-grade injuries, and was considered as UKA candidate; grades 3–4 were combined as high-grade injuries, and was considered as TKA candidate, and the surgical choice according to Recht grade was recorded.

**Table 1:** Cartilage injure classification: Recht grade by MRI and Outerbridge classification through intraoperative assessment

Grade	Recht grade	Outerbridge classification
0	Normal	Normal
1	Focal low signal in cartilage with smooth cartilage surface	Cartilage softening and edema or blisters on the surface
2	Defect < 50% cartilage thickness	Superficial ulcers and fibrosis < 1 cm
3	Defect ≥ 50% cartilage thickness but not down to subchondral bone	Deep ulcers ≥ 1 cm with crab-like changes
4	Full-thickness cartilage defect with exposure of subchondral bone	Full-thickness cartilage tear, exposed subchondral bone

### Intraoperative assessment

After making a medial parapatellar incision, the knee was flexed at 60°-90° so that the surgeon could observe the weight-bearing area cartilage of the lateral femoral condyle. Outerbridge grade [18] (Table 1) was the gold standard and was assessed by an experienced surgeon for intraoperative cartilage assessment. Slight cartilage injury of the lateral compartment did not affect the efficacy of UKA [19–21]. So, patients with Outerbridge grades 0–2 (non-high-grade injuries) underwent UKA, while patients with Outerbridge grades 3–4 (high-grade injuries) underwent TKA. Due to the incisional limitations of UKA, the cartilage of the tibial plateau was not exposed, so the cartilage quality in this part was not considered in our study.

### Statistical analysis

Imaging assessment and measurements were performed using UniSight. LJSW was measured in millimeters (mm). Descriptive histograms were used to examine the distribution of the variables. The Outerbridge grade was the gold standard, we compared it with valgus stress radiograph (LJSW) and MRI (Recht grade), and calculated the sensitivity, specificity, accuracy, positive predictive value (PPV) and negative predictive value (NPV) of LJSW and Recht grade in screening UKA. Then, we drew receiver operating characteristic (ROC) curves and calculated the area under the curve (AUC), respectively. *Z*-test was used to compare AUC.

Two blinded observers with more than three years’ experience in orthopedic image evaluating independently evaluated the Recht grade of MRI and LJSW of valgus stress radiograph, and the interobserver reliability was assessed. Intraobserver reliability was assessed after randomly selecting 20 images and repeated evaluation after two weeks. Excellent intraobserver and interobserver intraclass correlation coefficients were demonstrated in LJSW measurements by valgus stress radiograph (0.923 and 0.879) and in Recht grade by MRI (0.997 and 0.984).

*P*-value < 0.05 was considered significant. *P*-value < 0.01 was considered highly significant. Statistical tests were performed using SPSS 22.0 (SPSS Inc, Chicago, IL, USA).

###  Ethical approval statement

This prospective study was approved by the ethics committee. All of the procedures were performed in accordance with the Declaration of Helsinki. The study has been registered with the Chinese Clinical Trials Registry (ChiCTR2300072377).

## Results

### Demographics

We prospectively enrolled 125 patients with a total of 138 knees. The average age was 69 years old (range 51 to 89). Of 138 knees, 71 were left and 67 were right. Forty-five knees were observed with cartilage injuries (Outerbridge grade 1–4) in the lateral femoral weight-bearing area during surgery. With a comprehensive intraoperative evaluation, 120 knees with Outerbridge grades 0–2 (non-high-grade injuries) underwent UKAs, while 18 knees with Outerbridge grades 3–4 (high-grade injuries) underwent TKAs.

### Valgus stress radiographic LJSW

**Fig. 5:**
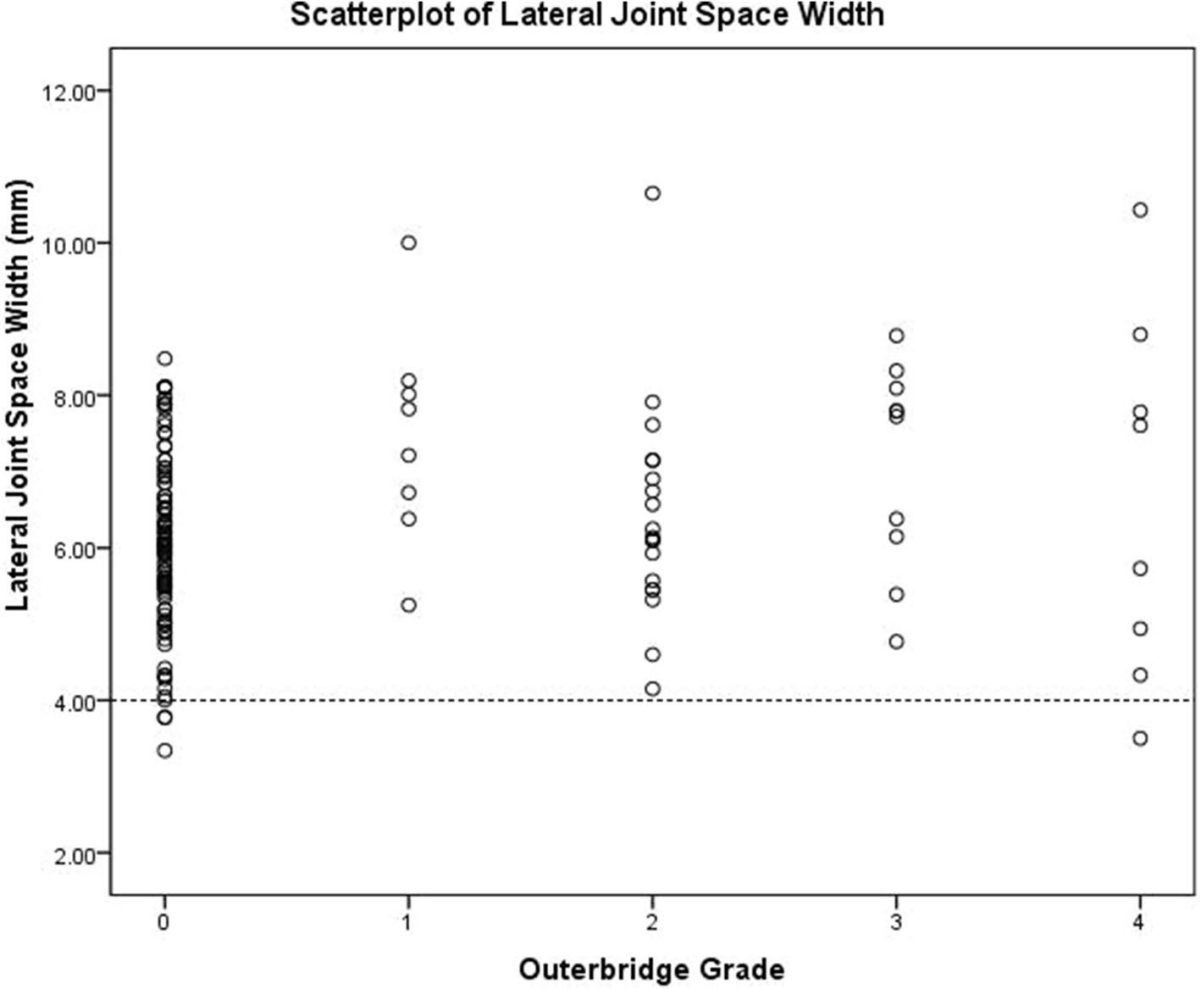
A scatterplot showing the distribution and relationship between LJSW on valgus stress radiograph and intraoperative Outerbridge grade of the lateral femoral weight-bearing area

The mean LJSW for all knees was 6.33 mm (range 3.34 to 10.65 mm). LJSW of five knees were ≤ 4 mm (four with Outerbridge grade 0 and only one with Outerbridge grade 4) (Fig. 5). The Outerbridge grade of 107 knees was greater than 0, and their LJSW was also ≥ 4 mm. Sixteen knees with high-grade injuries, while their LJSW was normal (Table 2).

**Table 2:** Distribution matrix of LJSW and Outerbridge grade of participants

LJSW (mm)	Outerbridge Grade		Total
	0–2 (non-high-grade injuries)	3–4 (high-grade injuries)	
> 4	117	16	133
≤ 4	4	1	5
Total	121	17	138

### MRI recht grade

The distribution of the Recht grade assessed by MRI and the Outerbridge grade assessed intraoperatively is shown in Table 3. Of the 45 knees with cartilage injuries, 21 (39.6%) knees were identified accurately by MRI. The sensitivity, specificity, accuracy, PPV, and NPV of MRI grading are shown in Table 4. The sensitivity of MRI in identification of cartilage injuries increased significantly from grade 1 to grade 4, while the specificity was greater than 90% for all grades. Accuracy and NPV showed good results (all ≥ 90%).

**Table 3:** Distribution matrix of Recht grade by MRI and Outerbridge grade of participants

Recht Grade	Outerbridge Grade					Total
	0	1	2	3	4	
0	84	7	5	0	1	97
1	4	1	1	0	0	6
2	4	0	8	0	0	12
3	0	0	4	5	0	9
4	1	0	1	5	7	14
Total	93	8	19	10	8	138

**Table 4:** The sensitivity, specificity, accuracy, positive predictive value, negative predictive value of MRI in detecting cartilage injuries

Outerbridge Grade	Sensitivity (%)	Specificity (%)	Accuracy (%)	PPV (%)	NPV (%)
1	12.5	96.2	91.3	16.7	94.7
2	42.1	96.6	89.1	66.7	91.3
3	50.0	96.9	93.5	55.6	96.1
4	87.5	94.6	94.2	50.0	99.2
Total	46.7	90.3	76.1	70.0	77.8

### Screening value for UKA of valgus stress radiograph and MRI

**Fig. 6:**
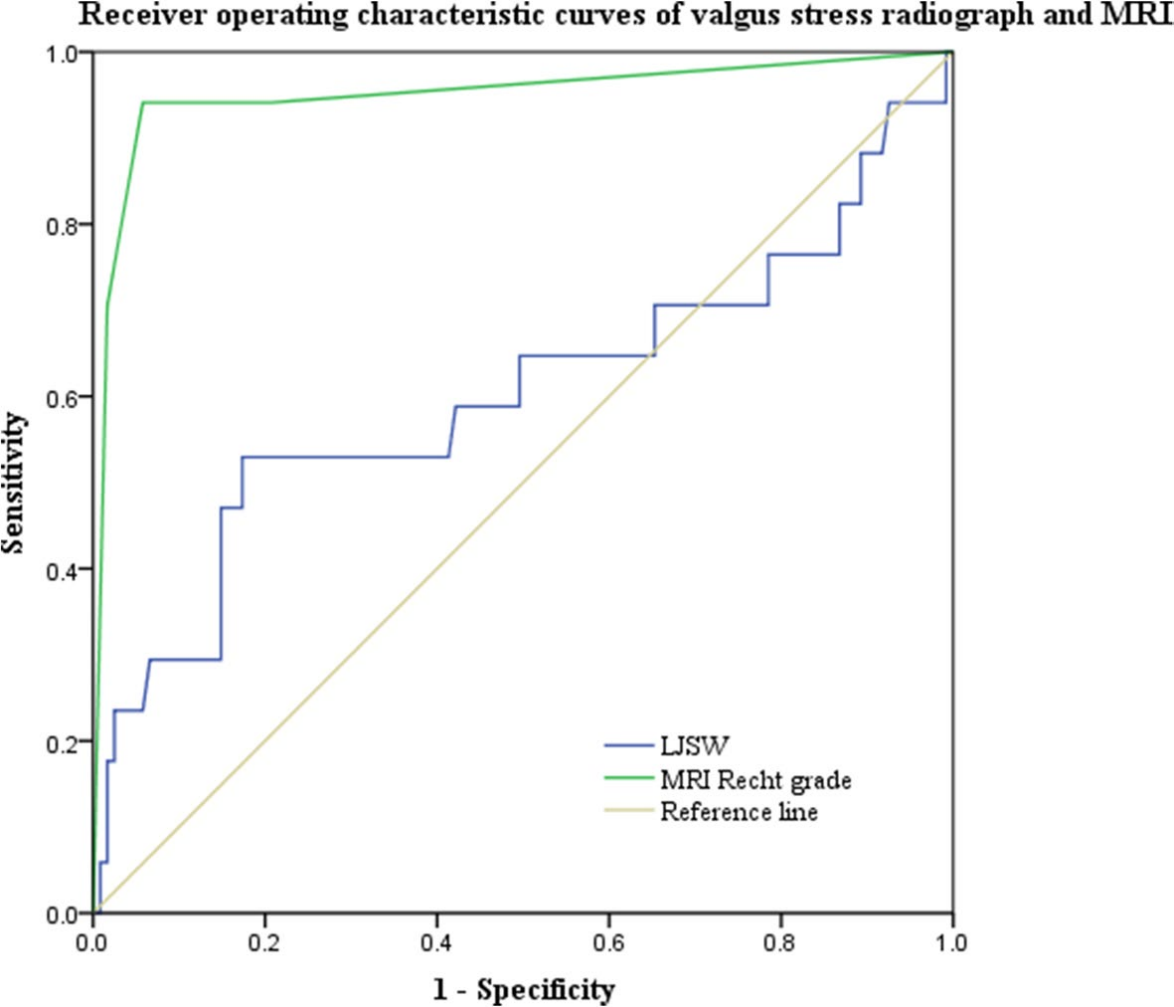
Receiver operating characteristic curves of MRI and lateral joint space measured on valgus stress radiograph in selecting UKA candidate. The difference between AUC of MRI (AUC = 0.950) and LJSW (AUC = 0.602) was 0.348 and was statistically significant (*P* = 0.001)

Both MRI and valgus stress radiograph had high sensitivity in screening UKA candidates. However, the specificity, accuracy, PPV and NPV of MRI were significantly higher than valgus stress radiograph (Table 5). ROC curves of MRI and valgus stress radiograph in screening UKA candidates are shown in Fig. 6. AUC of MRI was 0.950 and LJSW was 0.602 (Fig. 6). The difference between AUCs of MRI and LJSW was 0.348 and was significant (*P* = 0.001).

**Table 5:** The value of MRI and valgus stress radiograph in screening UKA candidates

	Sensitivity (%)	Specificity (%)	Accuracy (%)	PPV (%)	NPV (%)	ROC curve		
						AUC	95%*CI*	*P*
Valgus stress radiograph	96.7	5.9	85.5	88.0	20.0	0.602	0.425–0.778	0.176
MRI	95.0	94.4	94.9	99.1	73.9	0.950	0.877-1.000	< 0.001

## Discussion

The cartilage quality of the lateral compartment is the key to UKA survival [22, 23]. For patients with medial KOA, an accurate preoperative assessment of lateral compartment cartilage is critical for determining appropriate surgical options. Based on physical examination and weight-bearing radiograph, valgus stress radiograph and MRI are often used as auxiliary tools to diagnose the cartilage quality of lateral compartment. However, the comparison between valgus stress radiograph and MRI in evaluating cartilage quality in the lateral compartment prior to UKA has not been reported in the literature. Compared to valgus stress radiographic LJSW, we found that MRI has excellent value in identifying low- and high-grade injuries. That is, MRI is a reliable decision aid prior to UKA.

Joint space width measured on knee radiograph can monitor the progression and status of KOA [7]. Applying valgus stress to make the lateral-compartmental cartilage into close contact, valgus stress radiograph can be used to determine whether the cartilage thickness is normal in the lateral compartment [24]. The mean LJSW measured by the valgus stress radiograph in our study (6.33 mm) is consistent with the previously reported mean values (5.1-8.0 mm) [8, 13, 24, 25]. A study by Gibson reported a mean LJSW of 8.0 mm, but it only included 24 knees. He found that when LJSW is greater than 5 mm, the lateral compartment cartilage is intact [24]. Waldstein [13] found that LJSW on valgus stress radiograph can predict cartilage thickness accurately but not in relation to pathological cartilage degeneration. Another study’s measurements of 91 knees showed a mean LJSW of 5.4 mm, but it did not correlate well with the degree of lateral cartilage injury (*r* = 0.154, *P* = 0.146). In addition, all knees with Outerbridge grades 0–3 maintained LJSW at 4 mm or more; LJSW reduction only occurred in knees with Outerbridge grade 4 [8]. In our study, among the five knees with LJSW ≤ 4 mm, only one knee was Outerbridge grade 4, and the remaining four were grade 0. We think this is partially related to the dependence of LJSW on lateral meniscus function [26]. If the lateral meniscus is dislocated, LJSW will be reduced even if the cartilage is normal.

MRI is more sensitive in detecting early-stage changes in cartilage degeneration, particularly in T2-weighted imaging [27]. Accurate assessment of articular cartilage requires MRI with good spatial resolution to detect early cartilage lesions, high contrast to adequately display changes in cartilage signal intensity, and the distinction between cartilage, joint fluid, and subchondral bone [28].

Our study showed that MRI has good diagnostic value for the differentiation and grading of cartilage injuries, especially for high-grade injuries, which is consistent with the findings of Broderick [29] and Kawahara [10]. Studies on the diagnostic value of MRI vary greatly. Sensitivity ranged from 0 to 94% [11, 14–16, 27], while the specificity was higher than the sensitivity and at a high level. Based on sensitivity, Dutka [30] suggested that orthopaedic examination (51%) was more sensitive than MRI (32%). Most studies classified the cartilage changes as positive or negative and included a wide range of cartilage injury types. Few studies evaluated cartilage degeneration and made further classification. A study by Engelhardt [16] used 1.5 Tesla MRI to detect articular cartilage abnormality of KOA and reported sensitivity of 20% for grade 1, 52% for grade 2, 36% for grade 3, and 70% for grade 4. Kawahara [10] used 0.5 Tesla fast spin-echo MRI and reported sensitivity of 32% for grade 1, 72% for grade 2, 94% for grade 3, and 100% for grade 4. Another study found that using fast spin echo sequence, the sensitivity of grade 1 was 26%, grade 2 was 63%, grade 3 was 64%, and grade 4 was 77% [31]. This study used the 3 Tesla MRI and reported results consistent with our study - the sensitivity of MRI increased significantly with higher injury grades, especially for grade 3 and 4 injuries. In addition, these studies showed a significantly higher specificity than sensitivity, although their sample sizes were less than 75 cases [16, 29, 31].

Because low-grade cartilage injuries do not have much influence on the long-term effects of UKA [19–21], as long as image examination can distinguish between low-grade and high-grade injuries, it can become a suitable decision aid for UKA. Our study showed that the accuracy and specificity of MRI were much higher than valgus stress radiograph. In addition, when classifying the cartilage injuries as non-high-grade and high-grade injuries, MRI demonstrated higher sensitivity and specificity than when classifying them as Recht grades 0–4. This result is of great significance to the selection of UKA-appropriate candidates. Bredella [27] also classified cartilage injuries into early-stage (grades 1 and 2) and late-stage (grades 3 and 4), with 74% sensitivity and 85% specificity for early-stage and 85% sensitivity and 80% specificity for late-stage. It suggested that T2 fast spin echoes were more sensitive to early-stage injury and T2 fat-saturated fast spin echo sequences were more sensitive to end-stage injury. These findings indirectly confirmed that MRI could be used to select the appropriate candidates for UKA. However, Lombardi [32] proposed a different conclusion, arguing that preoperative MRI abnormalities do not affect the outcome of UKA. This different conclusion may be due to the bias arising from the excessive difference between the amount in the abnormal group (33 patients) and the normal group (967 patients), prolonged interval between MRI and surgery (within two years), and that the MRI images were interpreted by different radiologists rather than the same surgeon. In our study, MRI accurately identified 17 (94.4%) of the 18 knees undergoing TKA, while the valgus stress radiograph only identified 1 knee (5.9%). We observed that lateral weight-bearing area cartilage injury was more likely to be a focal injury or cartilage exfoliation rather than cartilage thinning or large-scale defects. Therefore, the cartilage that constitutes the space of the lateral compartment was still present, but the function was almost lost. However, the valgus stress radiograph can only evaluate the cartilage thickness, not the actual cartilage function. In contrast, focal injury or cartilage exfoliation can lead to joint fluid inflow into cartilage fissures with a significantly intensive signal on MRI. This strongly demonstrates the highly selective and exclusionary value of MRI for preoperative evaluation of UKA.

There are some limitations in this study. First, although the specific grading is not available to surgeons, they have access to the valgus stress radiograph and MRI preoperatively, which may impact intraoperative Outerbridge grading. Secondly, the valgus stress radiograph is obtained manually. Although strict and detailed requirements were made on how to take the radiograph, the instability caused by artificially applied valgus stress may cause bias. In addition, we did not sample intraoperatively for pathological grading, which would help identify the very early-stage cartilage changes. Finally, as the cartilage of the femoral weight-bearing area could only be exposed intraoperatively, we did not evaluate the cartilage of the lateral tibia plateau, which would also affect UKA survival. However, MRI demonstrated a very high diagnostic value for femoral cartilage, which may also apply to tibial cartilage.

## Conclusion

On the basis of physical examination and weight-bearing radiograph, compare to valgus stress radiograph, MRI has excellent diagnostic value for high-grade injuries and is suitable for selecting patients for medial UKA.

## Data Availability

The datasets used and analysed during the current study are available from the corresponding author on reasonable request.

## Abbreviations

AUC: Area under the curve
LJSW: Lateral joint space width
MRI: Magnetic resonance imaging
NPV: Negative predictive value
PPV: Positive predictive value
ROC: Receiver operating characteristic
TKA: Total knee arthroplasty
UKA: Unicompartmental knee arthroplasty
